# Neurophysiological processing of emotion and parenting interact to predict inhibited behavior: an affective-motivational framework

**DOI:** 10.3389/fnhum.2013.00326

**Published:** 2013-07-02

**Authors:** Ellen M. Kessel, Rebecca F. Huselid, Jennifer M. DeCicco, Tracy A. Dennis

**Affiliations:** ^1^Department of Psychology, Stony Brook UniversityStony Brook, NY, USA; ^2^Department of Psychology, Hunter College, The City University of New YorkNew York, NY, USA; ^3^Department of Psychology, Lafayette CollegeEaston, PA, USA

**Keywords:** inhibited behavior, emotional processing, parenting, late positive potential, children

## Abstract

Although inhibited behavior problems are prevalent in childhood, relatively little is known about the intrinsic and extrinsic factors that predict a child's ability to regulate inhibited behavior during fear- and anxiety-provoking tasks. Inhibited behavior may be linked to both disruptions in avoidance-related processing of aversive stimuli and in approach-related processing of appetitive stimuli, but previous findings are contradictory and rarely integrate consideration of the socialization context. The current exploratory study used a novel combination of neurophysiological and observation-based methods to examine whether a neurophysiological measure sensitive to approach- and avoidance-oriented emotional processing, the late positive potential (LPP), interacted with observed approach- (promotion) and avoidance- (prevention) oriented parenting practices to predict children's observed inhibited behavior. Participants were 5- to 7-year-old (*N* = 32) typically-developing children (*M* = 75.72 months, *SD* = 6.01). Electroencephalography was continuously recorded while children viewed aversive, appetitive, or neutral images, and the LPP was generated to each picture type separately. Promotion and prevention parenting were observed during an emotional challenge with the child. Child inhibited behavior was observed during a fear and a social evaluation task. As predicted, larger LPPs to aversive images predicted more inhibited behavior during both tasks, but only when parents demonstrated *low* promotion. In contrast, larger LPPs to appetitive images predicted less inhibited behavior during the social evaluative task, but only when parents demonstrated *high* promotion; children of high promotion parents showing smaller LPPs to appetitive images showed the greatest inhibition. Parent-child goodness-of-fit and the LPP as a neural biomarker for emotional processes related to inhibited behavior are discussed.

Social reticence and heightened fearful reactivity to novelty and threat are relatively stable aspects of behavior that emerge early in life (Kagan et al., 1988; Kagan and Snidman, 1991; Hane et al., 2008) and represent specific risk factors for a range of problems related to inhibited behavior and anxiety (Biederman et al., 2001; Pérez-Edgar and Fox, 2005; Kagan, 2008; Degnan et al., 2010). However, precious little is known about the intrinsic and extrinsic factors that predict a child's ability to regulate behavior during fear- and anxiety-provoking tasks. This question is particularly challenging given that signs of inhibited behavior show immense heterogeneity and are stable across development in only 10–15% of children (Kagan, 1994; Fox et al., 2001). Recent models highlight the interactive roles of child emotional processing sensitivities and the caregiving environment in predicting inhibited and anxious behavior in children (Fox et al., 2007; Murray et al., 2009; Schmidt and Miskovic, 2013), but empirical evidence remains scarce.

The goal of the current study was to use an affective-motivational framework to identify measures of emotional processing and parenting that may interact to influence a child's ability to regulate behavior during fear- and anxiety-provoking tasks. We target core motivational dimensions of approach and avoidance because they represent separable but interacting systems that are thought to organize patterns of biobehavioral self-regulation in children and adults (e.g., Fowles, 1994; Derryberry and Rothbart, 1997; Panksepp, 1998; Carver et al., 2000; Davidson, 2000; Gray and McNaughton, 2000) Approach reflects sensitivity to rewards, emotionally positive anticipation for pleasurable activities, and behavioral approach to novelty and challenge; in contrast, avoidance reflects sensitivity to potential threats, fear and shyness, and behavioral withdrawal and inhibition in response to novelty and challenge (Derryberry and Rothbart, 1997; Panksepp, 1998; Kagan, 1999; Carver, 2004). In a typically-developing group of children, we explored whether a neurophysiological measure of emotional processing, the late positive potential (LPP), in response to avoidance-oriented (aversive) and approach-oriented (appetitive) images interacts with avoidance- or approach-oriented parenting practices to predict the degree to which children show inhibited behavior. This exploratory research has the potential to identify a target biomarker and a target measure of caregiving relevant to individual differences in inhibited behavior, thus laying the groundwork for future, large-scale studies examining intrinsic and extrinsic mechanisms in the emergence of problems with behavioral inhibition and anxiety.

## Emotional processing and anxiety-related inhibited behavior

Across numerous studies, vigilance to and enhanced processing of aversive, fear-, and threat-relevant stimuli have been associated with anxiety (MacLeod et al., 1986; Vasey et al., 1996; Theall-Honey and Schmidt, 2006; Bar-Haim et al., 2007; Roy et al., 2008; Telzer et al., 2008; Waters et al., 2010) and have been used to explain which children at temperamental risk for anxiety go on to develop anxiety disorders (Pérez-Edgar et al., 2010, 2011). For example, Perez-Edgar and colleagues (2010) found that temperamental behavioral inhibition predicted social anxiety in adolescents, but primarily among those who evidenced biased attention to threat.

However, other studies suggest that anxious individuals show *reduced* processing of aversive or threat-relevant stimuli, suggesting attentional avoidance (Weierich et al., 2008; Bar-Haim et al., 2010). For example, in a recent large-scale community-based study, among children diagnosed with distress-related disorders (e.g., generalized anxiety disorder), high levels of internalizing symptoms predicted vigilance to angry faces, whereas among children diagnosed with social anxiety disorder, internalizing symptoms predicted avoidance of angry faces (Salum et al., 2013). These findings are consistent with models proposing that anxious individuals may show both vigilance and avoidance of threatening and aversive stimuli (Mogg et al., 2004; Weierich et al., 2008).

Compounding the complexity of this research, additional studies suggest that inhibited and anxious individuals show greater sensitivity not only to these avoidance-related aversive cues, but also to approach-related appetitive cues (Hardin et al., 2006; Bar-Haim et al., 2009; Helfinstein et al., 2011). For example, in one study, adolescents with a childhood history of inhibition, in comparison to those with no such history, showed greater striatal activation in anticipation of both monetary gain and loss (Guyer et al., 2006). Moreover, a childhood history of inhibition has also been associated with the presence of an anxiety disorder for adolescents demonstrating greater reactivity to high-incentive rewards (Pérez-Edgar et al., 2013). One interpretation of these results is that in anxiety-provoking tasks, strong approach motives may exacerbate approach-avoidance conflicts, leading to intensified fear and inhibition at the expense of approach inclinations (Asendorpf, 1990; Schmidt and Fox, 1994; McNaughton and Corr, 2004). Thus, increased processing of approach-related appetitive stimuli may indicate a specific affective sensitivity promoting inhibited behavior during fear- and anxiety-eliciting tasks (Helfinstein et al., 2012).

To examine whether processing of both aversive and appetitive stimuli is related to individual differences in inhibited behavior, the current study explored, in typically-developing children, whether a neurophysiological measures of emotional processing, the LPP, was systematically related to inhibited behavior in tasks designed to elicit fear and social-evaluative anxiety. This question represents a crucial first step in identifying whether the LPP is a viable candidate biomarker for affective vulnerability factors related to inhibition.

## Neurophysiological measures of emotional processing: the late positive potential

Disruptions in emotional processing are often covert and rapid, and thus might not be readily apparent in observable behavior (MacLeod et al., 1986; Bar-Haim et al., 2007). Moreover, high temporal sensitivity may be necessary for measuring both facilitation and avoidance of emotional processing, which may emerge at distinct time point along the emotional processing continuum (Amir et al., 1998; Mogg et al., 2004). Scalp-recorded event-related potentials (ERPs) derived from electroencephalography (EEG) are particularly well suited for this goal given their highly sensitive temporal specificity on the order of milliseconds. Moreover, stimulus-locked ERPs are relatively independent from behavioral response requirements, and are highly feasible for measuring brain processes across a range of age and clinical groups (Fox et al., 2005; Banaschewski and Brandeis, 2007).

Research using very early-emerging ERPs suggests that anxiety-related traits and disorders are associated with both facilitation and avoidance of aversive stimuli. For example, Mueller and colleagues (2009), using a dot probe task, found that individuals with social phobia evinced greater P1 amplitudes in response to angry compared to happy faces, indicative of early facilitation of attention, but reduced P1 amplitudes once the angry faces were replaced by probe stimuli, suggesting later avoidance. On the other hand, Jetha and colleagues (2012) showed that shy adults evidence *reduced* P1 amplitudes to fearful faces, whereas Kolassa and Miltner (2006) failed to find any association between social phobia and P1 amplitudes but did find increased face-specific N170 amplitudes in response to angry faces. Although these findings suggest that anxiety-related traits are linked to both enhanced emotional processing and avoidance very early in the processing stream, results are contradictory and cannot address the full time course of emotional processing.

The LPP, is a promising candidate ERP component for measuring individual differences in approach- and avoidance-related emotional processing. The LPP reflects facilitated attention to motivationally salient emotional vs. neutral stimuli in both children and adults (Keil et al., 2002; Schupp et al., 2004; Foti and Hajcak, 2008; Hajcak and Dennis, 2009; Kujawa et al., 2012; Solomon et al., 2012). Specifically, the amplitudes of the LPP are larger for emotional vs. neutral stimuli beginning around 250 or 300 ms after a stimulus is presented and extending throughout the course of picture processing as well as after picture offset (Hajcak and Olvet, 2008). The LPP combines very rapid temporal resolution on the order of milliseconds with the ability to measure sustained emotional processing of aversive and appetitive images over seconds. In terms of its scalp distribution, the LPP is topographically dynamic, tending to shift over time from posterior to relatively anterior regions (Solomon et al., 2012). Moreover, the LPP shows good to excellent reliability across trials (Moran et al., 2013). Despite subtle developmental differences in the LPP's latency and topography (Hajcak and Dennis, 2009; Kujawa et al., 2012; Solomon et al., 2012) that may result from brain maturation in regions involved in emotion regulation and cognitive control (Casey et al., 2000), preliminary evidence suggests that the LPP is also relatively stable over time (Kujawa et al., under review). Thus, the LPP is able to capture an extended time course of emotional processing (Moser et al., 2008; MacNamara and Hajcak, 2009, 2010; MacNamara et al., 2011) that may reflect stable individual differences in emotional processing. Moreover, previous research has shown that greater LPP amplitudes in response to aversive stimuli are associated with greater state anxiety in adults (Moser et al., 2008; MacNamara and Hajcak, 2009, 2010) and with greater trait anxiety in children (Decicco et al., 2012). No studies to date have examined whether individual differences in the LPP are related to observed inhibited behavior in children.

In the current study, our primary hypothesis was that enhanced processing of aversive stimuli measured via the LPP will predict greater inhibited behavior during fear- and social evaluative tasks. In addition, drawing on the anxiety literature documenting enhanced sensitivity to appetitive and reward-related cues (e.g., Pérez-Edgar et al., 2013), we tested the exploratory hypothesis that enhanced processing of appetitive stimuli would also be associated with greater inhibition. As discussed below, however, these associations should be moderated by caregiving context.

## The role of caregiving context

Given that individual differences in emotional processing may contribute to the ability to regulate behavior during fear- and anxiety-provoking situations, it is critical to examine extrinsic social factors, such as parenting, that shape patterns of emotional responding (Fox et al., 2007; Hane et al., 2008; Penela et al., 2012). Indeed, children showing temperamental negative affectivity may be more susceptible to the influence of parenting (Belsky and Pluess, 2009), in particular those aspects of parenting that serves to highlight approach or avoidance motives (Howes and Phillipsen, 1998; Hay et al., 2004; Dennis, 2006; Fox et al., 2007). Despite strong theoretical support for the idea that neurobiological factors influence developmental pathways to inhibition and anxiety *in conjunction* with social context, few studies have brought together these areas of research.

Mounting evidence suggests that specific patterns of parenting influence the expression of inhibited behavior, in particular via parenting's impact on emotional processing tendencies (Fox et al., 2005, 2007). Fox and colleagues (2007), in their Plasticity for Affective Neurocircuitry model, provide a framework for examining the role of environmental factors, such as parenting, in the developmental trajectory toward anxiety. They propose that the interplay between early caregiving environment and emotional processing of threat-relevant stimuli influence the link between temperament and later problems with anxiety and behavioral inhibition. In particular, this model posits that caregiving environments that highlight threat or fail to remediate a threat focus, such as low caregiver sensitivity or high caregiver intrusiveness (Ghera et al., 2006; Hane and Fox, 2006), exacerbate disrupted processing of threat-relevant stimuli and thus alter affective neurocircuitry in such a way that promotes and maintains anxiety-related behaviors in children.

This model focuses on caregiver sensitivity and intrusiveness, but does not articulate the possibility that parenting strategies that *directly* promote avoidance-related threat sensitivity or approach-related appetitive sensitivities may play a crucial role in the link between emotional processing tendencies and inhibited behaviors in anxiety-provoking circumstances. Based on motivational models of self-regulation (Higgins, 1997; Higgins and Silberman, 1998; Keller, 2008), our lab has developed an observation-based measure of parenting that reflects the degree to which parenting is characterized by behaviors that increase approach sensitivity (promotion parenting) by emphasizing accomplishment and the possibility of positive or desired outcomes, or by behaviors that increase avoidance and threat sensitivity (prevention parenting) by emphasizing safety, rules, and the need to avoid negative outcomes. For example, in one study (Dennis, 2006) levels of observed promotion parenting influenced whether child temperamental approach was associated with frustration and persistence during an emotional challenge.

Children showing greater emotional processing of aversive images in particular may benefit from high levels of promotion parenting because it fosters greater approach sensitivity by explicitly encouraging accomplishment, exploration and social participation (Higgins and Silberman, 1998). This “antidote” to enhanced avoidance-related emotional processing may ameliorate tendencies toward inhibited behavior, or even promote adaptive behavior when approach and avoidance motivations are in conflict (Asendorpf, 1990; Derryberry and Tucker, 2006; Hardin et al., 2006; Helfinstein et al., 2012; Schmidt and Miskovic, 2013). In contrast, prevention parenting, which highlights potential danger and threat, may exacerbate threat and avoidance-related emotional processing tendencies (Fox et al., 2007).

The notion that the effects of caregiving depend upon the transactions between child and parent characteristic, or goodness-of-fit, is a crucial concept here. In the current study, we examined goodness-of-fit in terms of whether the motivational fit between child emotional processing of aversive and appetitive stimuli (measured via the LPP) and promotion and prevention parenting predicts child inhibited behavior. We predicted that among children showing enhanced processing of aversive images, low levels of promotion and/or high levels of prevention would predict more inhibited behavior during fear- and anxiety-provoking tasks. Predictions concerning enhanced processing of appetitive stimuli are more difficult to generate given the lack of previous research on this topic. However, if high approach sensitivity exacerbates approach-avoidance conflicts during anxiety-provoking tasks (Asendorpf, 1990), leading to intensified inhibition, then one possibility is that if children showing enhanced processing of appetitive images experience *low* levels of promotion parenting, this reflects poor goodness-of-fit and engenders more approach-avoidance conflict and inhibited behavior.

## The current study

The study included typically-developing, early school-aged children (5- to 7-year-olds). The goal of the current study was to examine whether, in this normative group, enhanced processing of aversive and appetitive stimuli interacts with parenting that promotes approach or avoidance motivational tendencies to predict the regulation of behavior during fear- and anxiety-provoking tasks—specifically the degree to which children showed inhibition in response to these challenges. This study is novel in that it is among the first to use the LPP as a biomarker for biased emotional processing in relation to inhibited behavior, and the first to use an affective-motivational framework to conceptualize the interplay, or goodness-of-fit, between a neurophysiological measure of emotional processing and parenting that may be relevant to the emergence of problems with inhibited behavior and anxiety.

We tested the following two hypotheses: (1) Children showing larger LPP amplitudes to aversive vs. neutral images will show more inhibited behavior, but mainly when mothers show high prevention or low promotion; and (2) Children showing larger LPP amplitudes to appetitive vs. neutral images will show more inhibited behavior, but mainly when mothers show low promotion.

## Method

### Participants

Participants were 32 (19 males) typically developing school-aged children between the ages of five and seven (*M* = 75.72 months, *SD* = 6.01) and their caregivers. Parent-child dyads were recruited from in and around New York City. Our sample comprised of 10 Caucasian children, 13 African American children, two Asian American children, five Hispanic children and two children were reported as multiracial by their caregivers. Each child and caregiver spent ~3 h in the laboratory as part of a larger study on emotional development and was compensated $100 for their time. Additionally, children were given certificates of completion and astronaut ice cream at the end of their visits.

This study was derived from a larger study that yielded a previous publication examining the LPP in school-aged children (Solomon et al., 2012). This goal of this study was to test the neurodevelopmental question of whether, like adults, children at this age evidence larger LPP amplitudes to emotional vs. neutral images; this study did not examine the LPP in relation to parenting to predict inhibited behavior, the goal of the current study. Eighty-two percent of (*n* = 39) participants from the previous study were included in the current analyses. The selection criterion was the presence of observed parenting data, which was missing for seven children due to task refusal (three) and data loss due to poor or lost video recording (four).

### Procedures and materials

Upon arrival to the laboratory, an experimenter played a game with the children, while another experimenter obtained informed consent from the parents. Immediately following, verbal assent was obtained from the child. Children were subsequently escorted by an experimenter to another room to begin the EEG portion of the visit. While EEG was recorded from children, parents completed various questionnaires pertaining to their child's temperament and behavior. After the EEG recording was completed and children took a short break, children proceeded to complete the behavioral portion of the visit with their parents, including the black box, storytelling and wait task in addition to several behavioral tasks not included in the current study.

### Passive viewing procedure and stimuli

Once EEG setup was complete, children were moved to a dimly lit experiment booth equipped with a video camera and were instructed not to move or talk while passively viewing 90 images from the IAPS. Children were seated 65 cm from a 17″ computer monitor as images were presented in full screen and color using Presentation software (Version 2, Neurobehavioral Systems, Inc.; Albany, CA) on an IBM computer. The images were presented in a randomized order, and each stimulus was presented for 2000 ms with an inter-stimulus interval of 500 ms.

Images were 30 unpleasant^1^, 30 pleasant^2^, and 30 neutral^3^ pictures selected from the International Affective Picture System (IAPS; Lang et al., 2005). Unpleasant images are characterized by the IAPS developers as aversive: meaning to elicit affect related to defensive motivation, such as fear and disgust. In contrast, pleasant images are characterized as appetitive, in that they elicit affect related to approach motivation, such as joy, excitement, desire, or affiliation. The specific aversive images in the current study were chosen to reflect threat or potential threat (e.g., wreckage and war images) in a developmentally appropriate way. Aversive images had a mean valence of 3.32 (*SD* = 1.74) and a mean arousal of 5.79 (*SD* = 2.10). Appetitive images (e.g., food, babies, cuddly animals) had a mean valence of 7.45 (*SD* = 1.50) and a mean arousal of 4.76 (*SD* = 2.30). Neutral images (e.g., household and nature images) had a mean valence of 5.29 (*SD* = 0.74) and a mean arousal of 2.81 (*SD* = 0.65)^4^. Valence and arousal ratings are on a 9-point scale, with lower ratings for valence and arousal corresponding to more aversive and less arousing, respectively.

### Observed inhibited behavior

After EEG was recorded, inhibited behavior was measured during the black box and storytelling tasks, both adapted from the Laboratory Temperament Assessment Battery (LabTAB; Goldsmith et al., 1995).

The 2-min black box task was designed to elicit inhibited behavior to a novel and fear-inducing stimulus while in the presence of an adult. After an opaque black box with a covered opening on its side was placed on the center of the table, the experimenter neutrally told the child, “This is my special black box. There is something kind of scary inside. Would you like to put your hand in this hole to feel what is inside?” The task ended when the child reached his or her hand into the opaque box and removed the brightly colored soft squeezable ball covered in tentacles or after 2 min had passed. Inhibited behavior was measured as the latency (in seconds) before children placed their hand in the black box, with higher scores indicating greater inhibition.

The 7-min storytelling task was designed to elicit inhibited behavior related to social anxiety in response to the threat of criticism. Children were given a picture book and told that an assistant who was an expert on telling stories would listen to them tell a story and assign them a grade. After the child was finished telling his story, the experimenter praised him and gave him an “A+.” Inhibited behavior was quantified as the amount of time the child waited (latency score) before beginning storytelling, with higher scores indicating greater inhibition. Coders were trained to record latency using practice videotapes until reaching 80% agreement.

### Observed parenting and behavioral coding

Parenting was observed during a waiting task (WT; Carmichael-Olson et al., 1985). The WT is a parent-child task designed to both elicit child frustration as well as enable observation of parenting behaviors in response to child frustration. Parent-child dyads were alone in a room for 10 min after the experimenter handed the parent a clipboard of several papers to complete, gave the child a boring plastic toy and placed an attractively wrapped surprise on the table. The parent was previously instructed as soon as the experimenter left the room to tell the child, “This is a surprise for you, but you must wait until I finish my work to open it” (Cole et al., 2003). The parent was given no further instructions on how to interact with his or her child through the duration of the task. After the wait task was complete, the child was permitted to open and play with the wrapped yo-yo.

Parenting behavior focusing on reward or threat was coded using the Promotion/Prevention Parenting Coding System (Dennis and Cole, 2001; Dennis, 2006). Parenting behaviors and verbalizations were coded within 10-s epochs during the waiting task and were summed to create a total score. Parental behaviors that fit neither category were labeled non-codable (50.01% were coded as non-codable).

Promotion parenting focuses on the promotion of positive child behavior and orienting children toward potential reward. Examples include eliciting competent action (“Do you know what that is?”), encouraging compliance for a positive reason (“If you wait, you can open the present.”), guiding (“They're going to bring your snack in just a minute.”), commenting on the positive (“This won't take long.”), giving encouragement through affection and appreciation (“Great job.”) and maternal withdrawal of maternal positive reinforcement (I'm sad that you're not listening to me.”).

Prevention parenting focuses on child safety, the prevention of negative outcomes, and orienting children toward potential threat. Examples include eliciting appropriate behaviors and safety (“They asked you to wait.”), rewarding conformity with rules (“Thank you for not opening the present.”), prohibiting and intervening (“Listen I don't want you to… ”), encouraging compliance for a negative reason or rule (“ Because I said so.”), commenting on the negative (“Uh oh.”), and criticizing (“You're being bad.”). Two coders were trained to code promotion/prevention parenting by using practice videotapes until they reached 80% agreement. Then, inter-rater reliability using Cohen's Kappa was conducted to determine consistency among raters on the basis of 20% of the videos (7 videos), randomly chosen. The Kappa coefficient was 0.73, (*p* < 0.001), reflecting substantial agreement (Landis and Koch, 1977).

### EEG recording and data reduction

Using the Biosemi system (BioSemi; Amsterdam, NL), EEG activity was recorded continuously via 64 Ag/AgCl scalp electrodes embedded in an elasticized nylon cap based on the international 10/20 system. Eye movements were monitored by electro-oculogram (EOG) signals from electrodes placed 1 cm above and below the left eye (to measure vertical eye movements) and one cm on the outer edge of each eye (to measure horizontal eye movements). The EEG signal was preamplified at the electrode to improve the signal-to-noise ratio. EEG was recorded at a sampling rate of 512 Hz and amplified with a band pass of 0.16–100 Hz. The voltage from each active electrode was referenced online with respect to a common mode sense active electrode producing a monopolar (non-differential) channel. All data preparation after recording was conducted using Brain Vision Analyzer (Version 2.2, GmbH; Munich, DE). Data were re-referenced offline to an average mastoid reference and filtered with a high pass frequency of 0.1 Hz and a low pass frequency of 30 Hz. The EEG was segmented for each trial beginning 400 ms prior to picture onset and continuing for 2000 ms. Baseline correction was performed for each trial, using the 400 ms prior to picture onset.

EEG was corrected for blinks using independent components analysis. Artifacts were identified using the following criteria: any data with voltage steps exceeding 75 μV, changes within a segment that were greater than 200 μV, amplitude differences greater than ±120 μV within a segment, and activity lower than 0.2 μV per 100 ms were considered artifacts and excluded from analyses. Trials were also visually inspected for remaining artifacts. Data from individual channels containing artifacts were rejected on a trial-by-trial basis.

The LPP was measured as mean amplitudes for each picture type separately, in three time windows based on visual inspection of the data: early (300–700 ms), middle (700–1200 ms) and late (1200–2000 ms). Examining multiple time windows is particularly important because vigilance-avoidance patterns of processing aversive stimuli have been shown to vary over time (Holmes et al., 2008; Mueller et al., 2009), including studies of the LPP showing reduced LPPs to aversive stimuli among anxious individuals in *later* time windows (Weinberg and Hajcak, 2011). The LPP was calculated as the mean amplitude separately for each window in three broad regions. Regions were chosen based on visual inspection of the topographical distribution of the LPP (see Figure 2) and were consistent with previous findings regarding the diffuse scalp distribution of the LPP in children (Dennis and Hajcak, 2009). Regions were: posterior (PO4, PO8, O2, Oz, POz, PO3, PO7, and O1), central (C4, C6, CP6, Cz, CPz, C3, C5, and CP5), and anterior (FC4, F4, F6, Fpz, AFz, FC3, F3, and F5). Difference scores were generated for each four window/region combinations in which LPP amplitudes were maximal (e.g., posterior/early, central/middle, central/late, and anterior/late) to quantify the degree to which aversive or appetitive vs. neutral images generated larger LPPs (e.g., LPPs aversive—LPP neutral images). These difference scores were used as an index of the degree of emotional processing of aversive and appetitive images.

### Analytic plan

Interactions between parenting behavior (promotion or prevention) and each of the four LPP aversive –neutral difference and four LPP appetitive-neutral difference scores (e.g., early/posterior) predicting inhibitory behavior (Black Box and Storytelling tasks) were tested using Ordinary Least Squares multiple-regression interactions. For both dependent variables, two predictors (e.g., promotion parenting and LPP aversive-neutral difference scores) were entered in step one, and their interaction term in step two. Simple models were used to maximize power and ensure that sample size was over 30 and more than 10 cases per predictor were in each model^5^. A total of 16 regression models were estimated.

All variables in these analyses were screened first for univariate normality. Our storytelling latency variable was positively skewed (2.08) because of a few very inhibited children who delayed storytelling to the maximum time limit of 300 s. Kurtosis was also high (3.81) so violations of normality led us to do a square-root transformation (square root is taken of each score) which brought it within normal parameters (skew = 1.25, kurtosis = 0.79). This transformation was chosen because the data had no negative values or scores between 0 and 1. Also it produced a normal distribution without completely removing the inherent skew in the data which reflects variation in the inhibitory behavior that is of interest (Osborne, 2002). All other study variables were relatively normal with skewness and kurtosis indices less than ±2. The Mahalanobis Distance statistic was used to test multivariate normality and potential undue influence of outliers–cases with an unusual combination of scores on two or more variables. No cases were found to be significant multivariate outliers (with four predictors the critical value for the Mahal distance = 18.467), so follow-up analyses were not run. Predictor variables were centered (deviated from their mean) to reduce potential multicollinearity between interaction terms and their constituent variables. Significant interactions were probed following procedures described by Jaccard et al. (1990) and figures were created using high/low values one standard deviation above/below the mean.

## Results

### Descriptive statistics and correlations among study variables

**Figure 1:**
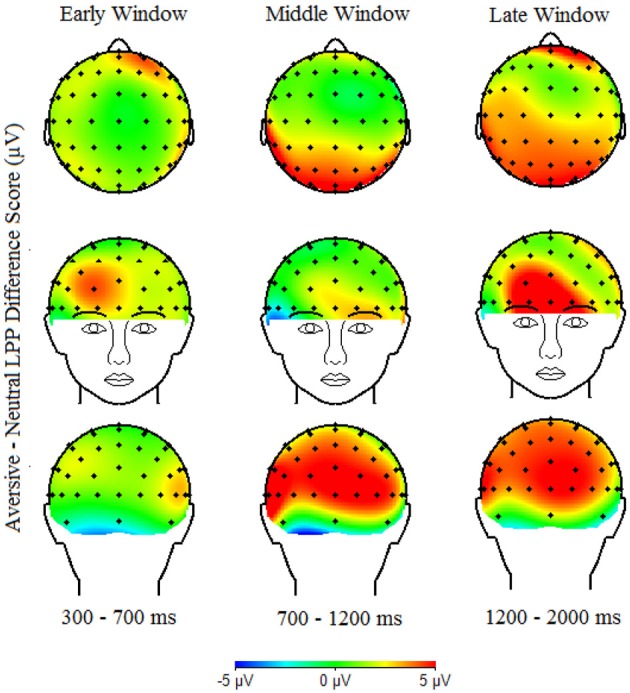
**Scalp topography of the LPP Aversive—Neutral difference score in the early (300–700 ms), middle (700–1200 ms) and late (1200–2000 ms) time windows**.

**Figure 2:**
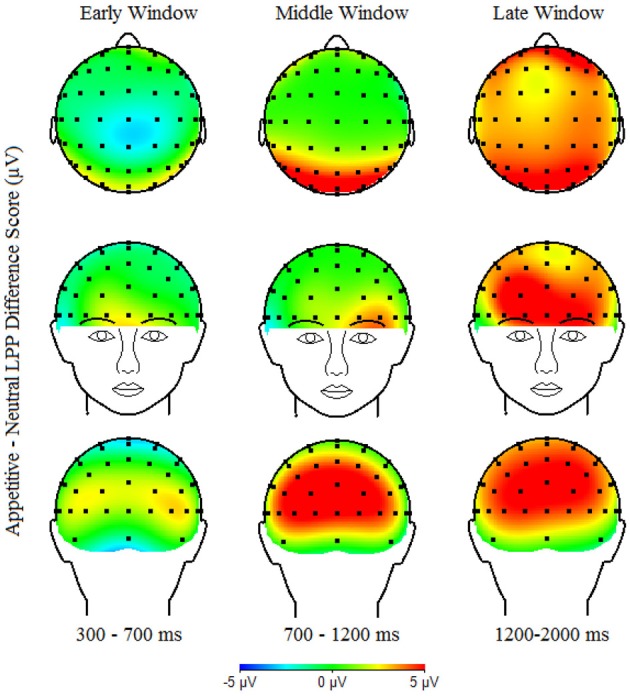
**Scalp topography of the LPP Appetitive—Neutral difference score in the early (300—700 ms), middle (700–1200 ms) and late (1200–2000 ms) time windows**.

**Figure 3:**
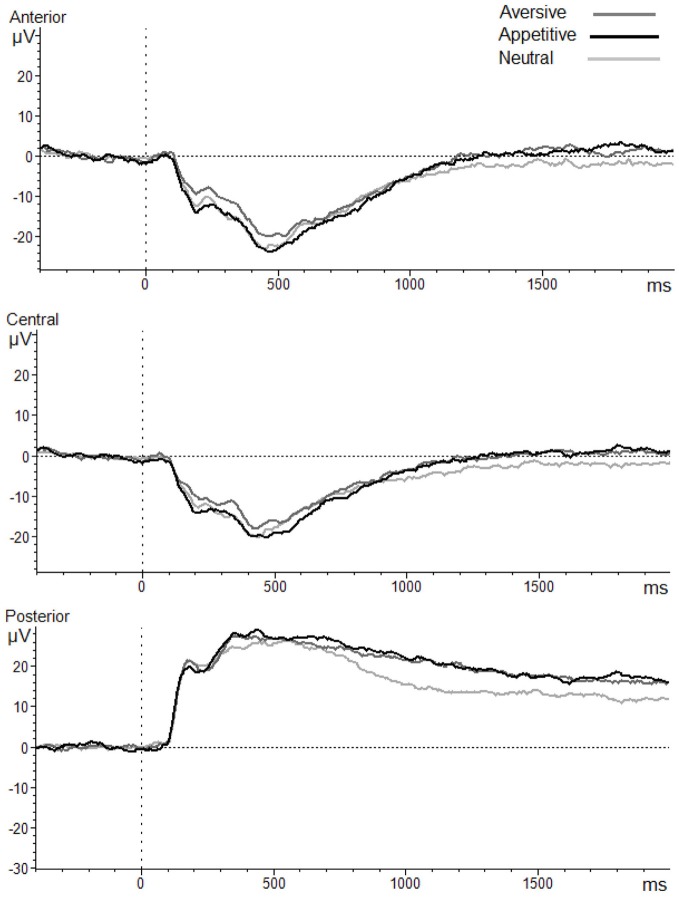
**LPP amplitudes in posterior, central and anterior regions during passively viewing aversive, appetitive, and neutral IAPS images**.

Table 1 presents descriptive statistics for observed maternal promotion and prevention during the waiting task, and observed child inhibited behavior during the black box and storytelling tasks. Child gender and ethnicity were not significantly associated with any study variables and are thus not included in analyses below. As can be seen in Figures 1, 2, which shows the scalp distribution of the LPP aversive—neutral and appetitive—neutral difference scores during each time window, the scalp topography of the LPP shifts from posterior to relatively anterior (central-frontal) regions over the duration of the LPP (300–2000 ms). Figure 3 shows the waveform for the LPP to aversive, appetitive, and neutral images. Bivariate correlations were conducted among observed maternal promotion and prevention during the waiting task, observed child inhibited behavior during the black box and storytelling tasks, and the LPP aversive-neutral and appetitive-neutral difference score at the early/posterior, middle/central, late/central, and late/anterior time-window/region.

**Table 1:** **Descriptive statistics**.

**Variable**	**Mean**	***SD***	**Range**
Promotion parenting	6.00	4.16	0.00–13.00
Prevention parenting	12.28	8.67	0.00–30.00
Inhibited behavior black box task	37.44	38.53	4.00–120.00
Inhibited behavior social storytelling task	56.13	80.61	1.00–300.00

Parenting and child inhibited behavior variables were not significantly intercorrelated, although inhibited behavior during each task was marginally positively correlated (*p* = 0.07). In contrast, LPPs were significantly positively intercorrelated. The multiple time-window/regions of the LPP aversive-neutral difference scores correlated with one another (significant *rs* ranged from 0.47 to 0.64). The same was true of the LPP appetitive-neutral difference scores (significant *rs* ranged from 0.37 to 0.89). Additionally, there were positive associations between LPP aversive-neutral and appetitive-neutral difference scores (significant *rs* ranged from 0.37 to 0.54). Given that inhibited behavior during each task was only marginally positively correlated and we believe that these two tasks tap into different dimensions of inhibited behavior, with the black box task eliciting fear and the storytelling task eliciting social anxiety, we examined each separately in analyses below.

### Emotion processing × parenting predicting inhibited behavior

**Figure 4:**
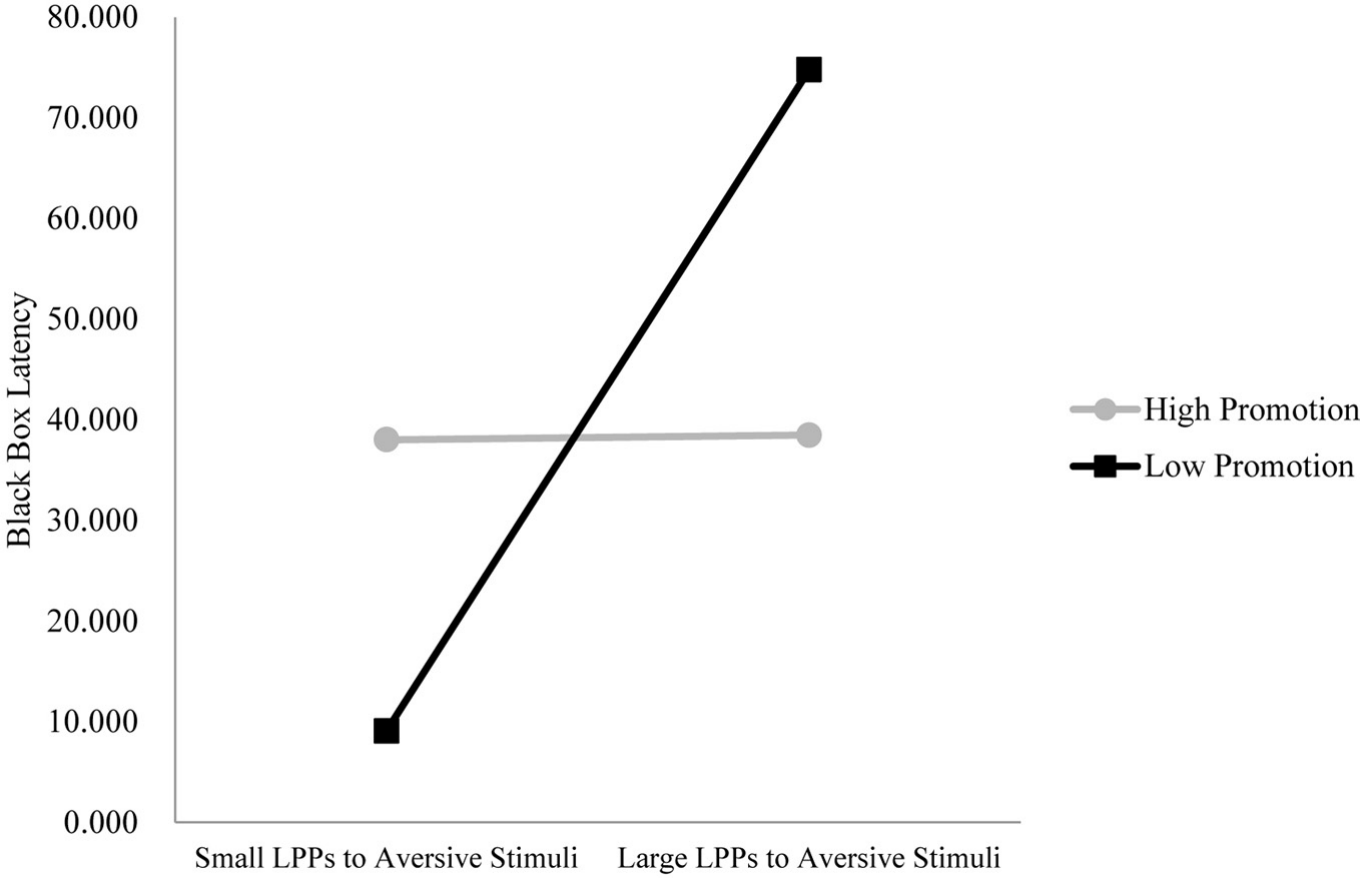
**LPPs to aversive stimuli interact with promotion parenting to predict inhibited behavior during the black box task**. *Note*: Longer latencies indicate more inhibited behavior. LPPs are quantified as the difference between LPP amplitudes to aversive minus LPPs to neutral images in the anterior region/late window. Model with LPP (anterior/late), Promotion Parenting, LPP × Promotion: Δ*R*^2^ = 0.186; unstandardized regression coefficients: High Promotion group *b* = 0.03, *t*_(28)_ = 0.02, *p* > 0.05; Low Promotion group *b* = 4.44, *t*_(28)_ = 2.63, *p* < 0.01.

**Figure 5:**
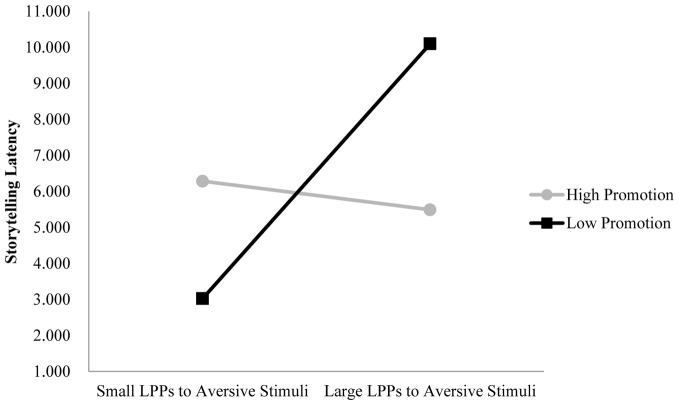
**LPPs to aversive stimuli interact with promotion parenting to predict inhibited behavior during the storytelling task**. *Note*: Longer latencies indicate more inhibited behavior. LPPs are quantified as the difference between LPP amplitudes to aversive minus LPPs to neutral images in the central/middle window. Model with LPP (central/middle), Promotion Parenting, LPP × Promotion: Δ*R*^2^ = 0.155; unstandardized regression coefficients: High Promotion group *b* = −0.07, *t*_(28)_ = −0.40, *p* > 0.05; Low Promotion group *b* = 0.67, *t*_(28)_ = 3.47. *p* < 0.01.

**Figure 6:**
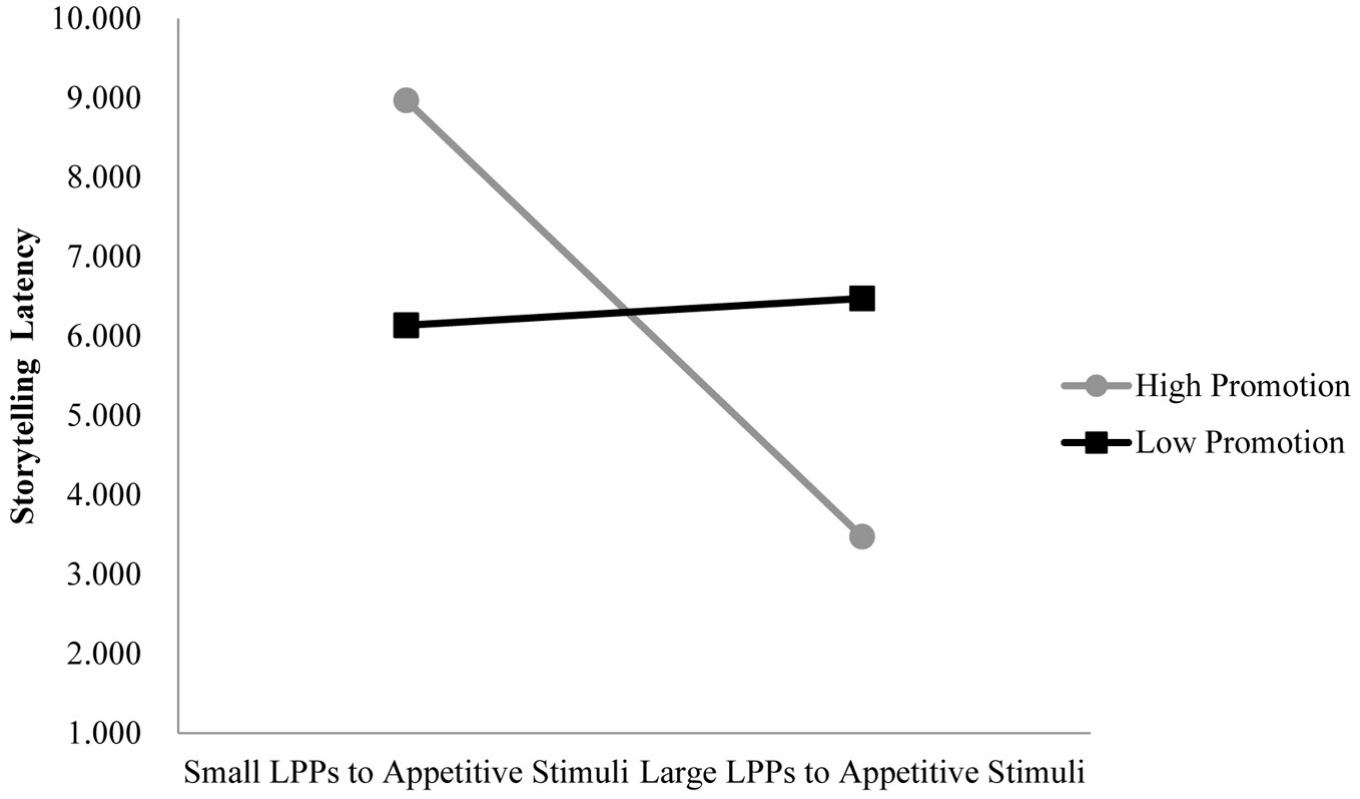
**LPPs to appetitive stimuli interact with promotion parenting to predict inhibited behavior during the storytelling task**. *Note*: Longer latencies indicate more inhibited behavior. LPPs are quantified as the difference between LPP amplitudes to appetitive minus LPPs to neutral images in the central/late window. Model with LPP (central/late), Promotion Parenting, LPP × Promotion: Δ*R*^2^ = 0.108; unstandardized regression coefficients: High Promotion group *b* = −0.37, *t*_(28)_ = −1.91, *p* < 0.05, one tailed; Low Promotion group *b* = 0.02, *t*_(28)_ = 0.12, *p* > 0.05.

We tested the specific hypothesis that children showing greater emotional processing of aversive stimuli (greater LPPs to aversive vs. neutral images) will show more inhibited behavior, but mainly when mothers show high prevention or low promotion parenting. We also tested the exploratory hypothesis that children showing greater emotional processing of appetitive stimuli who also have mothers showing low promotion will also show more inhibited behavior. Dependent variables were observed inhibited behavior during black box task and storytelling tasks. Figures 4, 5 show the significant interactions between LPPs (anterior/late and central/middle windows) to aversive stimuli and maternal promotion parenting on inhibited behavior during the black box task and the storytelling task, respectively (*t* = −2.67, *p* < 0.05, Δ*R*^2^ = 0.186 and *t* = −2.37, *p* < 0.05, Δ*R*^2^ = 0.155). Figure 6 shows the marginally significant interaction between the LPP (central/late window) to appetitive stimuli and maternal promotion parenting on inhibited behavior during the storytelling task, *t* = −1.86, *p* = 0.07, Δ*R*^2^ = 0.108 (see Table 2).

**Table 2:** **Standardized regression coefficients for interaction terms**.

	**Beta**	***t***	***p*-value**
**DEPENDENT VARIABLE—BLACK BOX LATENCY**	**F**_(3, 28)_ = 3.14* Δ*R*^2^ = 0.186^**^		
**Step 2**			
Promotion parenting	0.048	−0.29	0.78
Lpps to aversive stimuli (anterior/late)	0.429	2.4	0.02^*^
Promotion parenting × lpps to aversive stimuli (anterior/late)	−0.474	−2.64	0.01^**^
**DEPENDENT VARIABLE—STORYTELLING LATENCY**	F_(3, 28)_ = 2.71^†^ Δ*R*^2^ = 0.155^*^		
**Step 2**			
Promotion parenting	−0.08	−0.45	0.66
Lpps to aversive stimuli (central/middle)	0.34	2.02	0.05^*^
Promotion parenting × lpps to aversive stimuli (central/middle)	−0.4	−2.37	0.03^*^
**DEPENDENT VARIABLE—STORYTELLING LATENCY**	**F_(3, 28)_ = 1.36 Δ*R*^2^ = 0.108^†^**		
**Step 2**			
Promotion parenting	−0.01	−0.05	0.96
Lpps to appetitive stimuli (central/late)	−0.3	−1.47	0.15
Promotion parenting × lpps to appetitive stimuli (central/late)	−0.36	−1.86	0.07^†^

† p < 0.10,

* p ≤ 0.05,

** p ≤ 0.01.

As predicted, as preferential processing of aversive stimuli (larger LPPs) increased, observed behavior during the black box (*b* = 4.44) and storytelling tasks (*b* = 0.67) also becomes more inhibited, but only for children with relatively low promotion (approach-focused) parenting (see Figures 4, 5, respectively). For children with high promotion parenting, larger LPPs to aversive vs. neutral stimuli did not predict inhibited behavior during the black box task (*b* = 0.03) nor during the storytelling task (*b* = −0.07). Interestingly, it was the children with smaller LPPs to aversive stimuli and low promotion parenting that show the *least* inhibited behavior. Prevention parenting did not significantly interact with LPP measures of aversive image processing to predict inhibited behavior.

In contrast to our predictions, as preferential processing of appetitive vs. neutral stimuli (smaller LPPs) *decreased*, inhibition during the storytelling task increased (*b* = −0.37), but only for children with relatively high promotion (approach-focused) parents (see Figure 6). For children of parents showing low promotion, individual differences in LPPs to appetitive vs. neutral stimuli did not predict inhibited behavior during the storytelling task (*b* = 0.23). Moreover, as seen in Figure 6, it is the children with larger LPPs to appetitive stimuli with parents showing high promotion that show the *least* inhibited behavior.

## Discussion

The goal of the current study was to use an affective-motivational framework to identify measures of emotional processing and parenting that interact to influence a child's ability to regulate inhibited behavior during fear- and anxiety-provoking tasks. We explored whether the LPP, as a highly sensitive measure of emotional processing, could capture individual differences in the processing of approach-related (appetitive) and avoidance-related (aversive) stimuli that predicted the degree of inhibited behavior during fear and anxiety-related emotional challenges *in interaction* with avoidance- or approach-oriented parenting practices. Consistent with hypotheses, we found that children showing larger LPPs to aversive images also showed more inhibited behavior during both tasks, but only when parents demonstrated *low* promotion. In contrast, larger LPPs to appetitive images predicted less inhibited behavior during the social evaluative task, but only when parents demonstrated *high* promotion. Interestingly, those children of high promotion parents who also evidenced smaller LPPs to appetitive images showed the greatest inhibition. Results suggest that emotional processing of both appetitive and aversive stimuli should be considered when examining inhibited behavior. Results also suggest that it is crucial to further investigate how parenting that highlights approach and avoidance may be important social contexts in which to examine a child's emotional processing sensitivities, and their role in risk and resilience.

Findings of the current study capitalize on the use of both neurophysiological and observation-based measures to examine the interplay among intrinsic and extrinsic processes that predict the expression of inhibited behavior during emotional challenges that trigger fear and anxiety associated with social evaluation. To our knowledge, this was the first study to provide evidence to suggest that the LPP, when examined within the social context of parenting, may be a useful measure of emotion processing sensitivities in future studies examining the developmental trajectory of inhibited behavior and risk for anxious pathology.

As predicted, children showing enhanced processing of aversive stimuli also showed more inhibited behavior during both tasks, but only for children whose mothers demonstrated low levels of approach-focused promotion parenting. Interestingly, for children of mothers with high levels of promotion parenting, increasing LPP amplitudes to aversive images did not predict change in inhibited behavior. These findings highlight that high promotion parenting may serve a protective role for children who show enhanced avoidance-related processing of aversive stimuli – these children's behaviors are indistinguishable from children showing less emotional processing of aversive stimuli. Moreover, findings suggest that *reduced* opportunities to interact with caregivers in ways that could counteract avoidance-motivated affect—rather than increased opportunities to interact in ways that promote avoidance sensitivity (prevention parenting)—influenced children's abilities to regulate behavior in anxiety-related contexts. If children who show greater neurocognitive sensitivity to aversive stimuli have parents who do *not* highlight approach-related goals, this could exacerbate processing of aversive and threat-related stimuli, reduce ability to detect appetitive cues or cues for safety, and alter affective neurocircuitry accordingly to promote or maintain inhibition in children (Fox et al., 2007; Schmidt and Miskovic, 2013). Taken together, these results suggest that parents who show relatively low levels of promotion with children who show greater emotional processing of aversive stimuli may miss opportunities to bolster children's abilities to effectively regulate behavior when confronted with fear- and anxiety-related challenges.

Exploratory analyses also revealed that children showing enhanced processing of appetitive stimuli also showed *less* inhibited behavior during the storytelling task, but only for children whose mothers demonstrated high levels of promotion. For children of mothers with low levels of promotion, increasing LPP amplitudes to appetitive images did not predict change in inhibited behavior (the inverse was true for findings with aversive image processing reported above). Moreover, we found, counter to prediction, that children of high promotion parents who showed *less* processing of appetitive images showed the most inhibited behavior. This effect is puzzling, but one possibility is that the poor fit between a child showing low approach-related emotional processing and a parent showing high approach-related promotion parenting leads to less effective regulation during emotional challenges. Mirroring these results, Dennis (2006) found that the degree to which there was poor goodness-of-fit between high parental promotion and low child temperamental approach reactivity predicted whether a child evidenced both increased frustration and decreased persistence during lab-based tasks.

In the current study, the fact that appetitive picture processing effects only emerged in the social-evaluative storytelling task may relate to a conflict between task demands and motivational drive. That is, the motive to obtain positive social feedback is a strong approach-related motive, which could be in conflict with blunted appetitive emotional processing tendencies indicated by reduced LPPs to appetitive images. High levels of promotion parenting attempting to motivate a child's behavior with approach cues to which a child may be relatively insensitive may fail to appropriately scaffold a child's self-regulatory abilities. In contrast, for children who evidenced enhanced appetitive image processing, high promotion parenting may be an advantageous parenting practice to socialize children to effectively tackle achievement-oriented task demands when confronted with social-evaluative threat.

Taken together, findings underscore the possibility that promotion parenting is a social context that influences links between child emotional processing and inhibited behavior in typically-developing children. These results could thus set the stage for future research on the impact of motivationally-distinct patterns of parenting on positive outcomes in children at risk for problems with behavioral inhibition and anxiety (Belsky and Pluess, 2009). Of note, while promotion parenting interacted in distinct ways with LPP measures of aversive and appetitive picture processing, it did so at later stages of processing (the middle and late windows). This hints at the possibility that later-emerging and perhaps more effortful attentional processes are more sensitive to both costs and benefits of the socialization context (Dennis, 2006).

In addition, we found that promotion, but not prevention parenting, influenced whether emotional processing sensitivities influenced children's behavior during both the fear task (black box) and the social evaluative task (storytelling task). It may be that in this typically-developing group of children, the beneficial presence of approach-focused promotion parenting may be particularly important for predicting the expression of inhibition in response to fear and anxiety (Shechner et al., 2011). Future studies should measure sensitivities to both aversive and appetitive stimuli in the context of parenting when examining predictors of inhibited behavior during fear- and anxiety-inducing tasks. It is important to note that, given the greater frequency of observed prevention parenting, it is possible that some elements of the waiting task, such as concerns of compliance in a lab setting, were more likely to elicit prevention-focused parenting (although promotion parenting is also used to promote compliance; Higgins and Silberman, 1998). This is consistent with a previous study documenting greater frequency of prevention vs. promotion parenting in the waiting task (Dennis, 2006). If prevention parenting was preferentially elicited, this might have reduced our ability to detect subtle individual differences in this aspect of parenting, thus reducing predictive power.

In interpreting findings, we must consider that the current study differed from others in several important ways. First, an important methodological difference is that previous studies examining emotional processing or attention to emotion, particularly to threat, typically generate scores based on reaction times on a task involving attentional competition between threat and neutral stimuli, such as the dot probe or emotional Stroop (Bar-Haim et al., 2007). In contrast, in the current study, the LPP was generated in response to passive viewing of individual images with no task demands, and thus reflect performance-independent aspects of emotional processing. Indeed, in one study using an emotional interruption task in children age 8–13, the LPP was not consistently associated with behavioral responses (Kujawa et al., 2012).

Another important methodological difference was that the previous studies use a range of stimuli to measure emotional processing tendencies, most notably human faces, threat-relevant words, and, in the case of appetitive processing, rewards. In the current study, stimuli were taken from the IAPS, which reflect general aversive and appetitive affective dimensions, rather than being specific to reward or threat (although a large percentage of the IAPS selected in this study (87%) were specifically threat-related, such as images of threatening animals, angry human faces, guns pointed in the direction of the viewer). Moreover, some appetitive images in the present study (e.g., cute, furry animals and babies) were relatively low salience and arousal compared to studies using reward, erotica, or other such stimuli, many of which are not developmentally appropriate. Overall, however, IAPS may be more evocative and have more robust effects on both behavior and electrocortical activity compared to face (Kujawa et al., 2012) or word stimuli typically used to measure biased attention. Thus, the relatively high salience of the IAPS images used in the current study may have strengthened measurement of individual differences in emotional processing.

Limitations of this current research study include a relatively small sample size, which restrict the statistical power of our analysis, although we did meet sample size requirements to test for interactions. Additionally, we did not include any self-report data on the children's subjective ratings of both the valence and the arousal level of the IAPS images, given that in previous studies in our lab, children were unable to reliably rate the images (Derryberry and Rothbart, 1997). Thus, we are unable to determine the degree to which the children found the aversive images to be threatening, although previous research using these images shows that like adults, children perceive these images as aversive and arousing (Sharp et al., 2006). Since this is a normative group of children, results are inconclusive in terms of the utility of the LPP for measuring emotional processing sensitivities in clinically anxious and inhibited children. This is a crucial direction for future research, but the current study is an important first step in pursuit of this goal.

The current study is the first study how the LPP as a measure of attention to aversive and appetitive stimuli interacts with the socialization context to predict inhibited behavior. This question is particularly important for the target age group, school-aged children, which is a developmental period during which behavioral inhibition may trigger a cascade of biopsychosocial processes that create risk for later anxious psychopathology (Fox et al., 2005). Taken together, results suggest that the LPP holds promise as a biomarker for biased emotional processing of aversive and appetitive stimuli which may shape the developmental trajectory of inhibition, and that parenting that is motivationally-relevant is an important social context in which to examine this development. Future research should test this model in the context of pediatric anxiety, tracking whether individual differences in the LPP in response to aversive and appetitive stimuli and parental focus on approach and avoidance predict change and continuity in anxiety symptoms and atypical behavioral inhibition over time.

## Author note

This research was supported in part by grants from the National Institutes of Mental Health grant 5K01MH075764 and National Institute of General Medical Sciences Grant 5S06GM060654 awarded to Tracy A. Dennis. This research was also made possible by Grant RR03037 form the National Center for Research Resources (NCRR), a component of the National Institutes of Health.

### Conflict of interest statement

The authors declare that the research was conducted in the absence of any commercial or financial relationships that could be construed as a potential conflict of interest.

## Footnotes

^1^The IAPS numbers for aversive images were 1050, 1120, 1201, 1300, 1321, 1930, 2120, 2130, 2688, 2780, 2810, 2900, 3022, 3230, 3280, 5970, 6190, 6300, 6370, 7380, 9050, 9250, 9421, 9470, 9480, 9490, 9582, 9594, 9600, 9611.

^2^The IAPS numbers for appetitive images were 1460, 1463, 1601, 1610, 1710, 1750, 1811, 1920, 1999, 2070, 2091, 2165, 2224, 2311, 2340, 2345, 2791, 4603, 5831, 7325, 7330, 7400, 7502, 8031, 8330, 8380, 8461, 8490, 8496, 8620.

^3^The IAPS numbers for neutral images were 5220, 5711, 5740, 5750, 5800, 5820, 7000, 7002, 7004, 7006, 7009, 7010, 7025, 7031, 7035, 7041, 7050, 7080, 7090, 7100, 7140, 7150, 7175, 7190, 7224, 7233, 7235, 7236, 7595, 7950.

^4^Of the stimuli included in this study, 15 unpleasant, 8 pleasant, and 6 neutral images have normative ratings obtained by the IAPS developers from children aged seven to nine using the same rating system as adults and are as follows for valence and arousal respectively: Unpleasant (*M* = 3.74, *SD* = 1.22; *M* = 6.04, *SD* = 1.05), Pleasant (*M* = 8.27, *SD* = 0.84; *M* = 6.04, *SD* = 0.69), and Neutral (*M* = 5.90, *SD* = 0.18; *M* = 2.91, *SD* = 0.13). We did not select all child-normed stimuli because the current study was part of a larger study initiated when children were aged 5–6 (outside the norm age group) and stimuli were selected for age- and task-appropriateness rather than norms.

^5^Most experts recommend a minimum sample size at least 30, and at least 8 cases per predictor in multiple regression. We did meet these minimum standards with a sample size of 32 and no more than 3 terms in each equation. Although a larger sample size would be preferred for testing interactions. The most obvious limitation created by a small sample size is the lack of power to find hypothesized effects and an inflated type II error rate. Thus, the ability to find moderator effects is attenuated. These theoretically-driven predictions are testing joint effects of context and psychophysiology not previously tested. We believe this is currently the largest data set available to test moderating effects of temperament, parenting and LLP, and that regression analyses provide the best test of the predicted linear interactions between continuous predictors.

## References

[B1] Amir N. Foa E. B. Coles M. E. (1998). Automatic activation and strategic avoidance of threat-relevant information in social phobia. J. Abnorm. Psychol. 107, 285–290 10.1037/0021-843X.107.2.285 9604557

[B2] Asendorpf J. (1990). Beyond social withdrawal: shyness, unsociability, and peer avoidance. Hum. Dev. 33, 250–259 10.1159/000276522

[B3] Banaschewski T. Brandeis D. (2007). Annotation: what electrical brain activity tells us about brain function that other techniques cannot tell us - a child psychiatric perspective. J. Child Psychol. Psychiatry 48, 415–435 10.1111/j.1469-7610.2006.01681.x 17501723

[B4] Bar-Haim Y. Fox N. A. Benson B. Guyer A. E. Williams A. Nelson E. E. (2009). Neural correlates of reward processing in adolescents with a history of inhibited temperament. Psychol. Sci. 20, 1009–1018 10.1111/j.1467-9280.2009.02401.x 19594857 PMC2785902

[B5] Bar-Haim Y. Holoshitz Y. Eldar S. Muller D. Charney D. S. Pine D. S. (2010). Life-threatening danger and suppression of attention bias to threat. Am. J. Psychiatry 167, 694–698 10.1176/appi.ajp.2009.09070956 20395400

[B6] Bar-Haim Y. Lamy D. Pergamin L. Bakermans-Kranenburg M. J. Van IJzendoorn M. H. (2007). Threat-related attentional bias in anxious and nonanxious individuals: a meta-analytic study. Psychol. Bull. 133, 1–24 10.1037/0033-2909.133.1.1 17201568

[B7] Belsky J. Pluess M. (2009). Beyond diathesis stress: differential susceptibility to environmental influences. Psychol. Bull. 135, 885–908 10.1037/a0017376 19883141

[B8] Biederman J. Hirshfeld-Becker D. R. Rosenbaum J. F. Hérot C. Friedman D. Snidman N. (2001). Further evidence of association between behavioral inhibition and social anxiety in children. Am. J. Psychiatry 158, 1673–1679 10.1176/appi.ajp.158.10.1673 11579001

[B9] Casey B. J. Giedd J. N. Thomas K. M. (2000). Structural and functional brain development and its relation to cognitive development. Biol. Psychol. 54, 241–257 10.1016/S0301-0511(00)00058-2 11035225

[B10] Carmichael-Olson H. Greenberg M. Slough N. (1985). Coding Manual for the Wait Task. Seattle, WA: Department of Psychology, University of Washington

[B10c] Carver C. S. (2004). Negative affects deriving from the behavioral approach system. Emotion 4, 3–22 10.1037/1528-3542.4.1.3 15053723

[B10a] Carver C. S. Sutton S. K. Scheier M. F. (2000). Action, emotion, and personality: emerging conceptual integration. Pers. Soc. Psychol. Bull. 26, 741–751 10.1177/0146167200268008

[B10b] Davidson R. J. (2000). Affective style, psychopathology, and resilience: brain mechanisms and plasticity. Am. Psychol. 55, 1196–1214 10.1037/0003-066X.55.11.1196 11280935

[B11] Cole P. M. Teti L. O. Zahn C. (2003). Mutual emotion regulation and the stability of conduct problems between preschool and early school age. Dev. Psychopathol. 15, 1–18 10.1017/S0954579403000014 12848432

[B12] Decicco J. M. Solomon B. Dennis T. A. (2012). Neural correlates of cognitive reappraisal in children: an ERP study. Dev. Cogn. Neurosci. 2, 79–80 10.1016/j.dcn.2011.05.009 22163262 PMC3234882

[B13] Degnan K. A. Almas A. N. Fox N. A. (2010). Temperament and the environment in the etiology of childhood anxiety. J. Child Psychol. Psychiatry 51, 497–517 10.1111/j.1469-7610.2010.02228.x 20158575 PMC2884043

[B14] Dennis T. (2006). Emotional self-regulation in preschoolers: the interplay of child approach reactivity, parenting, and control capacities. Dev. Psychol. 42, 84–97 10.1037/0012-1649.42.1.84 16420120

[B14aa] Dennis T.A. Hajcak G. (2009). The late positive potential: a neurophysiological marker for emotion regulation in children. J. Child Psychol. Psychiatry 50, 1373–1383 10.1111/j.1469-7610.2009.02168.x 19754501 PMC3019134

[B15] Dennis T. A. Cole P. M. (2001). Promotion/Prevention Parenting Coding System. State College, PA: The Pennsylvania State University

[B14a] Derryberry D. Rothbart M. K. (1997). Reactive and effortful processes in the organization of temperament. Develop. Psychopathol. 9, 633–652 10.1017/S0954579497001375 9448999

[B16] Derryberry D. Tucker D. M. (2006). Motivation, self-regulation and self-organization, in Developmental Psychopathology, 2nd Edn Vol. 3, eds Cohen D. Cicchetti D. (New York, NY: Wiley and Sons), 502–531

[B17] Foti D. Hajcak G. (2008). Deconstructing reappraisal: descriptions preceding arousing pictures modulate the subsequent neural response. J. Cogn. Neurosci. 20, 977–988 10.1162/jocn.2008.20066 18211235

[B17a] Fowles D. C. (1994). A motivational theory of psychopathology, in Nebraska Symposium on Motivation. Integrated Views of Motivation and Emotion, Vol. 41, ed Spaulding W. (Lincoln: University of Nebraska Press), 181–228 7739747

[B18] Fox N. A. Hane A. A. Pine D. S. (2007). Plasticity for affective neurocircuitry: how the environment affects gene expression. Curr. Dir. Psychol. Sci. 16, 1–5 10.1111/j.1467-8721.2007.00464.x

[B19] Fox N. A. Henderson H. A. Marshall P. J. Nichols K. E. Ghera M. M. (2005). Behavioral inhibition: linking biology and behavior within a developmental framework. Annu. Rev. Psychol. 56, 235–262 10.1146/annurev.psych.55.090902.141532 15709935

[B20] Fox N. A. Henderson H. A. Rubin K. H. Calkins S. D. Schmidt L. A. (2001). Continuity and discontinuity of behavioral inhibition and exuberance: psychophysiological and behavioral influences across the first four years of life. Child Dev. 72, 1–21 10.1111/1467-8624.00262 11280472

[B21] Ghera M. M. Hane A. A. Malesa E. E. Fox N. A. (2006). The role of infant soothability in the relation between infant negativity and maternal sensitivity. Infant Behav. Dev. 29, 289–293 10.1016/j.infbeh.2005.09.003 17138284

[B22] Goldsmith H. H. Reilly J. Lemery K. Longley S. Prescott A. (1995). The Laboratory Temperament Assessment Battery Preschool Version. Madison, WI: University of Wisconsin

[B22a] Gray J. A. McNaughton N. (2000). The Neuropsychology of Anxiety, 2nd Edn. New York, NY: Oxford University Press

[B23] Guyer A. E. Nelson E. E. Perez-Edgar K. Hardin M. G. Roberson-Nay R. Monk C. S. (2006). Striatal functional alteration in adolescents characterized by early childhood behavioral inhibition. J. Neurosci. 26, 6399–6405 10.1523/JNEUROSCI.0666-06.2006 16775126 PMC6674047

[B24] Hajcak G. Dennis T. A. (2009). Brain potentials during affective picture processing in children. Biol. Psychol. 80, 333–338 10.1016/j.biopsycho.2008.11.006 19103249 PMC2656402

[B25] Hajcak G. Olvet D. M. (2008). The persistence of attention to emotion: brain potentials during and after picture presentation. Emotion 8, 250–255 10.1037/1528-3542.8.2.250 18410198

[B26] Hane A. A. Cheah C. Rubin K. H. Fox N. A. (2008). The role of maternal behavior in the relation between shyness and social reticence in early childhood and social withdrawal in middle childhood. Soc. Dev. 17, 795–811 10.1111/j.1467-9507.2008.00481.x

[B27] Hane A. A. Fox N. A. (2006). Ordinary variations in maternal caregiving influence human infants' stress reactivity. Psychol. Sci. 17, 550–556 10.1111/j.1467-9280.2006.01742.x 16771807

[B28] Hane A. A. Fox N. A. Henderson H. A. Marshall P. J. (2008). Behavioral reactivity and approach-withdrawal bias in infancy. Dev. Psychol. 44, 1491–1496 10.1037/a0012855 18793079 PMC2575804

[B29] Hardin M. G. Perez-Edgar K. Guyer A. E. Pine D. S. Fox N. A. Ernst M. (2006). Reward and punishment sensitivity in shy and non-shy adults: relations between social and motivated behavior. Pers. Individ. Dif. 40, 699–711 10.1016/j.paid.2005.08.010 19718273 PMC2733521

[B30] Hay D. F. Payne A. Chadwick A. (2004). Peer relations in childhood. J. Child Psychol. Psychiatry 45, 84–108 10.1046/j.0021-9630.2003.00308.x 14959804

[B31] Helfinstein S. M. Benson B. Perez-Edgar K. Bar-Haim Y. Detloff A. Pine D. S. (2011). Striatal responses to negative monetary outcomes differ between temperamentally inhibited and non-inhibited adolescents. Neuropsychologia 49, 479–485 10.1016/j.neuropsychologia.2010.12.015 21167189 PMC3065071

[B32] Helfinstein S. M. Fox N. A. Pine D. S. (2012). Approach-withdrawal and the role of the striatum in the temperament of behavioral inhibition. Dev. Psychol. 48, 815–826 10.1037/a0026402 22148946

[B33] Higgins E. T. (1997). Beyond pleasure and pain. Am. Psychol. 52, 1280–1300 10.1037/0003-066X.52.12.1280 9414606

[B34] Higgins E. T. Silberman I. (1998). Development of regulatory focus: promotion and prevention as ways of living, in Motivation and self-regulation across the life span, ed Dweck C. S. (New York, NY: Cambridge University Press), 78–113 10.1017/CBO9780511527869.005

[B35] Holmes A. Nielsen M. Green S. (2008). Effects of anxiety on the processing of fearful and happy faces: an event-related potential study. Biol. Psychol. 77, 159–173 10.1016/j.biopsycho.2007.10.003 18022310

[B36] Howes C. Phillipsen L. (1998). Continuity in children's relationships with peers. Soc. Dev. 7, 340–349 10.1111/1467-9507.000711488492

[B37] Jaccard J. Wan C. K. Turrisi R. (1990). The detection of interpretation of interaction effects between continuous variables in multiple regressions. Multivariate Behav. Res. 25, 467–478 10.1207/s15327906mbr2504_4 26820822

[B38] Jetha M. K. Zheng X. Schmidt L. A. Segalowitz S. J. (2012). Shyness and the first 100 ms of emotional face processing Shyness and the first 100 ms of emotional. Soc. Neurosci. 7, 37–41 10.1080/17470919.2011.581539 21777107

[B39] Kagan J. (1994). Galen's Prophecy. New York: Basic Books

[B39a] Kagan J. (1999). The concept of behavioral inhibition, in Extreme Fear, Shyness, and Social Phobia: Origins, Biological Mechanisms, and Clinical Outcomes. Series in Affective Science, eds Schmidt L. A. Schulkin J. (New York, NY: Oxford University Press), 3–13

[B40] Kagan J. (2008). Behavioral inhibition as a risk factor for psychopathology, in Child and Adolescent Psychopathology, eds Beauchaine T. P. Hinshaw S. P. (Hoboken, NJ: John Wiley and Sons), 157–179

[B41] Kagan J. Reznick J. S. Snidman N. (1988). Biological bases of childhood shyness. Science 240, 167–171 10.1126/science.3353713 3353713

[B42] Kagan J. Snidman N. (1991). Temperamental factors in human development. Am. Psychol. 46, 856–862 10.1037/0003-066X.46.8.856 1928938

[B43] Keil A. Bradley M. M. Hauk O. Rockstroh B. Elbert T. Lang P. J. (2002). Large-scale neural correlates of affective picture processing. Psychophysiology 39, 641–649 10.1111/1469-8986.3950641 12236331

[B44] Keller J. (2008). On the development of regulatory focus: the role of parenting styles. Eur. J. Soc. Psychol. 38, 354–364 10.1002/ejsp.460

[B45] Kolassa I. Miltner W. (2006). Psychophysiological correlates of face processing in social phobia. Brain Res. 1118, 130–141 10.1016/j.brainres.2006.08.019 16970928

[B46] Kujawa A. Klein D. N. Hajcak G. (2012). Electrocortical reactivity to emotional images and faces in middle childhood to early adolescence. Dev. Cogn. Neurosci. 2, 458–467 10.1016/j.dcn.2012.03.005 22521707 PMC3404214

[B48] Landis J. R. Koch G. G. (1977). The measurement of observer agreement for categorical data. Biometrics 33, 159–174 843571

[B49] Lang P. Bradley M. M. Cuthbert B. N. (2005). International Affective Picture System (IAPS): Instruction Manual for Affective Ratings. Gainesville, FL: The Center for Reseach in Psychophysiology, University of Florida

[B50] MacLeod C. Mathews A. Tata P. (1986). Attentional bias in emotional disorders. J. Abnorm. Psychol. 95, 15–20 10.1037/0021-843X.95.1.15 3700842

[B51] MacNamara A. Ferri J. Hajcak G. (2011). Working memory load reduces the late positive potential and this effect is attenuated with increasing anxiety. Cogn. Affect. Behav. Neurosci. 11, 321–331 10.3758/s13415-011-0036-z 21556695

[B52] MacNamara A. Hajcak G. (2009). Anxiety and spatial attention moderate the electrocortical response to aversive pictures. Neuropsychologia 47, 2975–2980 10.1016/j.neuropsychologia.2009.06.026 19576234

[B53] MacNamara A. Hajcak G. (2010). Distinct electrocortical and behavioral evidence for increased attention to threat in generalized anxiety disorder. Depress. Anxiety 27, 234–243 10.1002/da.20679 20196100

[B54] McNaughton N. Corr P. J. (2004). A two-dimensional neuropsychology of defense: fear/anxiety and defensive distance. Neurosci. Biobehav. Rev. 28, 285–305 10.1016/j.neubiorev.2004.03.005 15225972

[B55] Mogg K. Bradley B. Miles F. Dixon R. (2004). Time course of attentional bias for threat scenes: testing the vigilance-avoidance hypothesis. Cogn. Emot. 18, 689–700 10.1080/02699930341000158

[B56] Moran T. P. Jendrusina A. A. Moser J. S. (2013). The psychometric properties of the late. positive potential during emotion processing and regulation. Brain Res. [Epub ahead of print]. 10.1016/j.brainres.2013.04.018 23603408

[B57] Moser J. S. Huppert J. D. Duval E. Simons R. F. (2008). Face processing biases in social anxiety: an electrophysiological study. Biol. Psychol. 78, 93–103 18353522 10.1016/j.biopsycho.2008.01.005

[B58] Mueller E. M. Hofmann S. G. Santesso D. L. Meuret A. E. Bitran S. Pizzagalli D. (2009). Electrophysiological evidence of attentional biases in social anxiety disorder. Psychol. Med. 39, 1141–1152 10.1017/S0033291708004820 19079826 PMC3204217

[B59] Murray L. Creswell C. Cooper P. J. (2009). The development of anxiety disorders in childhood: an integrative review. Psychol. Med. 39, 1413–1423 19215631 10.1017/S0033291709005157

[B59a] Panksepp J. (1998). Affective Neuroscience: The Foundations of Human and Animal Emotions. New York, NY: Oxford University Press

[B60] Penela E. C. Henderson H. a Hane A. A. Ghera M. M. Fox N. A. (2012). Maternal caregiving moderates the relation between temperamental fear and social behavior with peers. Infancy 17, 715–730 23355798 10.1111/j.1532-7078.2012.00114.x PMC3551274

[B61] Pérez-Edgar K. Bar-Haim Y. McDermott J. M. Chronis-Tuscano A. Pine D. S. Fox N. A. (2010). Attention biases to threat and behavioral inhibition in early childhood shape adolescent social withdrawal. Emotion 10, 349–357 10.1037/a0018486 20515224 PMC3614079

[B62] Pérez-Edgar K. Fox N. A. (2005). Temperament and anxiety disorders. Child Adolesc. Psychiatr. Clin. N. Am. 14, 681–706, viii. 16171698 10.1016/j.chc.2005.05.008

[B63] Pérez-Edgar K. Hardee J. Guyer A. E. Benson B. Nelson E. Gorodetsky E. (2013). DRD4 and striatal modulation of the link between childhood behavioral inhibition and adolescent anxiety. Soc. Cogn. Affect. Neurosci. [Epub ahead of print]. 10.1093/scan/nst001 23314010 PMC3989122

[B64] Pérez-Edgar K. Reeb-Sutherland B. C. McDermott J. M. White L. K. Henderson H. A. Degnan K. A. (2011). Attention biases to threat link behavioral inhibition to social withdrawal over time in very young children. J. Abnorm. Child Psychol. 39, 885–895 10.1007/s10802-011-9495-5 21318555 PMC3756613

[B65] Roy A. K. Vasa R. A. Bruck M. Mogg K. Bradley B. P. Sweeney M. (2008). Attention bias toward threat in pediatric anxiety disorders. J. Am. Acad. Child Adolesc. Psychiatry 47, 1189–1196 18698266 10.1097/CHI.0b013e3181825ace PMC2783849

[B66] Salum G. A. Mogg K. Bradley B. P. Gadelha A. Pan P. Tamanaha A. C. (2013). Threat bias in attention orienting: evidence of specificity in a large community-based study. Psychol. Med. 43, 1–13 10.1017/S0033291712001651 22850475

[B67] Schmidt L. A. Miskovic V. (2013). A new perspective on temperamental shyness: differential susceptibility to endoenvironmental influences. Soc. Pers. Psychol. Compass 7, 141–157 10.1111/spc3.12014

[B68] Schupp H. Cuthbert B. Bradley M. Hillman C. Hamm A. Lang P. (2004). Brain processes in emotional perception: motivated attention. Cogn. Emot. 18, 593–611 10.1080/02699930341000239

[B69] Sharp C. Van Goozen S. Goodyer I. (2006). Children's subjective emotional reactivity to affective pictures: gender differences and their antisocial correlates in an unselected sample of 7-11-year-olds. J. Child Psychol. Psychiatry 47, 143–150 10.1111/j.1469-7610.2005.01464.x 16423145

[B70] Shechner T. Britton J. C. Pérez-Edgar K. Bar-Haim Y. Ernst M. Fox N. A. (2011). Attention biases, anxiety, and development: toward or away from threats or rewards. Depress. Anxiety 29, 281–294 10.1002/da.20914 22170764 PMC3489173

[B71] Schmidt L. A. Fox N. A. (1994). Patterns of cortical electrophysiology and autonomic activity in adults shyness and sociability. Biol. Psychol. 38, 183–198 10.1016/0301-0511(94)90038-8 7873702

[B72] Solomon B. Decicco J. M. Dennis T. A. (2012). Emotional picture processing in children: An ERP study. Dev. Cogn. Neurosci. 2, 110–119 10.1016/j.dcn.2011.04.002 22163263 PMC3234883

[B73] Telzer E. H. Mogg K. Bradley B. P. Mai X. Ernst M. Pine D. S. (2008). Relationship between trait anxiety, prefrontal cortex, and attention bias to angry faces in children and adolescents. Biol. Psychol. 79, 216–222 18599179 10.1016/j.biopsycho.2008.05.004 PMC2574721

[B73a] Theall-Honey L. A. Schmidt L. A. (2006). Do temperamentally shy children process emotion differently than nonshy and gender differences in reticent preschoolers. Develop. Psychobiol. 48, 187–196 10.1002/dev.20133 16568410

[B75] Vasey M. W. El-Hag N. Daleiden E. L. (1996). Anxiety and the processing of emotionally threatening stimuli: distinctive patterns of selective attention among high- and low-test-anxious children. Child Dev. 67, 1173–1185 10.2307/1131886 8706516

[B76] Waters A. M. Henry J. Mogg K. Bradley B. P. Pine D. S. (2010). Attentional bias towards angry faces in childhood anxiety disorders. J. Behav. Ther. Exp. Psychiatry 41, 158–164 20060097 10.1016/j.jbtep.2009.12.001

[B77] Weierich M. R. Treat T. A. Hollingworth A. (2008). Theories and measurement of visual attentional processing in anxiety. Cogn. Emot. 22, 985–1018 10.1080/02699930701597601

[B78] Weinberg A. Hajcak G. (2011). Electrocortical evidence for vigilance-avoidance in Generalized Anxiety Disorder. Psychophysiology 48, 842–851 10.1111/j.1469-8986.2010.01149.x 21073479

